# Study TPX-100-5: intra-articular TPX-100 significantly delays pathological bone shape change and stabilizes cartilage in moderate to severe bilateral knee OA

**DOI:** 10.1186/s13075-021-02622-8

**Published:** 2021-09-17

**Authors:** Dawn McGuire, Michael Bowes, Alan Brett, Neil A. Segal, Meghan Miller, David Rosen, Yoshinari Kumagai

**Affiliations:** 1 OrthoTrophix, Foster City, CA 94404 USA; 2 Imorphics, Manchester, UK; 3 University of Kansas Medical Center, Kansas City, KS USA

**Keywords:** *B*-score, Bone shape, DMOAD, Machine learning, Osteoarthritis, TPX-100

## Abstract

**Background:**

TPX-100, a promotor of osteoblast and chondroblast differentiation, is a potential osteoarthritis (OA) therapy. This retrospective study compared MRI 3D femoral bone shape changes (*B*-scores) after intra-articular TPX-100 or placebo and analyzed the relationship between cartilage thickness and bone shape change over 12 months.

**Methods:**

One hundred and four participants with bilateral moderate to severe knee cartilage defects were randomized to receive TPX-100 (200 mg) or placebo. Each subject’s contralateral placebo-treated knee served as a paired internal control. After MRI quality control, 78/93 subjects (84%; 156 knees) were analyzed for quantitative femoral *B*-score and cartilage thickness. All analyses were performed centrally, blind to treatment assignment and clinical data.

**Results:**

TPX-100-treated knees (*n* = 78) demonstrated a statistically significant decrease in pathologic bone shape change compared with placebo-treated knees at 6 and 12 months: 0.0298 (95% C.I. − 0.037, 0.097) vs 0.1246 (95% C.I. 0.067, 0.182) (*P* = 0.02), and 0.0856 (95% C.I. 0.013, 0.158) vs. 0.1969 (95% C.I. 0.123, 0.271) (*P* = 0.01), respectively. The correlation between bone shape change and medial and total tibiofemoral cartilage thickness changes at 12 months was statistically significant in TPX-100-treated knees (*P* < 0.01).

**Conclusions:**

This is the first report of a potential therapy demonstrating a significant effect on bone shape measured by *B*-score in knee OA. These data, in combination with previously reported statistically significant and clinically meaningful improvements in WOMAC physical function versus placebo, support TPX-100 as a candidate for disease modification in knee OA.

**Trial registration:**

NIH ClinicalTrials.gov, NCT01925261. Registered 15 August 2013

**Supplementary Information:**

The online version contains supplementary material available at 10.1186/s13075-021-02622-8.

## Background

TPX-100 is a 23-amino acid peptide derived from Matrix Extracellular Phosphoglycoprotein (MEPE), a small integrin-binding ligand N-linked glycoprotein (*SIBLING*) *family* member. MEPE is highly expressed by osteocytes, is downregulated in osteoarthritis, and may play a role in osteoarthritic bone remodeling [1–3].

TPX-100 has been shown to induce articular cartilage formation in vitro and in vivo. Pre-clinically, TPX-100 (AC-100) was administered by 4 weekly intra-articular (IA) injections in a standardized full-thickness chondral defect goat model. After 6 months, TPX-100 demonstrated robust articular (hyaline) cartilage formation and increased Type II collagen compared with vehicle-treated controls as demonstrated by increased type II collagen (immunostaining) [4].

**Fig. 1:**
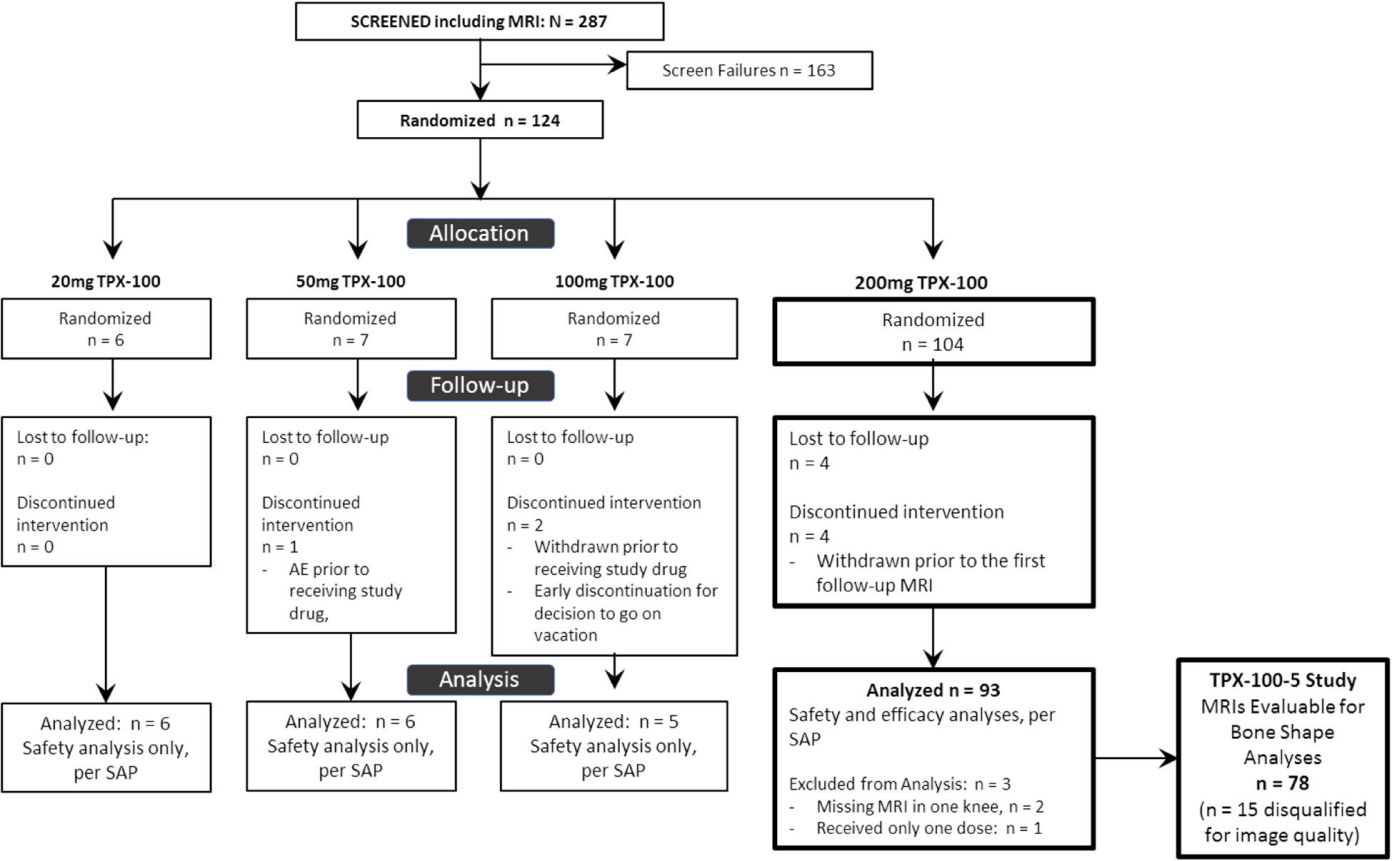
CONSORT diagram for the TPX-100-1 and TPX-100-5 studies

In humans, a phase II randomized, double-blind, placebo-controlled, 12-month trial of TPX-100 (NCT01925261) has been completed to evaluate safety, tolerability, and efficacy of IA TPX-100 in subjects with bilateral (ICRS grades 2–3) patellofemoral (PF) cartilage defects, with or without tibiofemoral cartilage defects [5, 6]. Each subject’s contralateral placebo-treated knee served as a paired internal control, intended to control for effects of age, sex, weight, genetic factors, and activity levels on outcome measures. Subjects were screened clinically and with MRI, inclusion criteria were applied for patellofemoral osteoarthritis (PFOA) severity (grades 2–3), and agnostic for osteoarthritis (OA) severity in other joint compartments. Synovitis and meniscal damage were among the exclusion criteria for enrollment. The pre-selected primary efficacy outcome measure in this trial was the 6-month change in patellar cartilage thickness as measured using standardized magnetic resonance imaging (MRI) in TPX-100-treated knees compared with placebo-exposed knees (see Fig. 1; CONSORT diagram). Briefly, part A of the study evaluated safety of 4 once-weekly IA doses of TPX-100 in sequential dose cohorts (25, 50, 100, and 200 mg/injection) of 6–9 subjects, with progression to the next dose following Safety Review Committee approval. All doses were reasonably safe and well tolerated, and the highest dose, 200 mg/injection, was selected for part B of the study for evaluation of efficacy and safety. Of the 118 subjects enrolled for parts A and B, 93 subjects received 4 injections of 200 mg TPX-100 and had baseline MRIs with at least one follow-up scan. Per the statistical analysis plan, these subjects made up the primary analysis population. Efficacy outcome measures included MRI cartilage measures (6 and 12 months) and patient-reported outcomes (WOMAC, KOOS, and NRS for pain at 3, 6, and 12 months). The primary efficacy outcome measure was patellofemoral cartilage change compared to baseline at six months in TPX-100 treated versus control knees. There were no significant treatment differences in the primary efficacy outcome measure at either 6 or 12 months. Semi-quantitative MRI analysis by central readers blind to clinical data and treatment assignment demonstrated that only 14% of knees had measurable patellar cartilage thickness changes, limiting study power for this outcome. MRI-based MOAKS evaluation, including cartilage defects, meniscal pathology, and Hoffa’s synovitis, did not show differences at baseline or follow-up between TPX-100-treated knees and placebo-exposed knees, and there were no significant within-knee changes in bone marrow lesions at 6 or 12 months. In contrast, patient benefit, measured by WOMAC and KOOS scores, was statistically significant and clinically meaningful in favor of TPX-100-treated knees at 6 months compared with placebo, with robust functional benefits sustained through the end of the study at 12 months [3]. Post hoc analyses revealed that 68 (73%) of subjects had, in addition to the bilateral patellofemoral cartilage defects for which they were enrolled, moderate to severe (ICRS 2–4) bilateral tibiofemoral cartilage defects. In these subjects, sustained, statistically significant, and clinically meaningful clinical benefits in favor of TPX-100-treated knees were observed, nearly identical to those in the whole population [6].

The present study (TPX-100-5) was designed as a retrospective MRI study to investigate femoral bone shape change at 6 and 12 months after TPX-100 or placebo administration and to analyze relationships between cartilage thickness and femoral bone shape change at 6 and 12 months after TPX-100 or placebo administration.

Bone shape change, measured by MRI, has been shown to predict radiographic onset of OA [7], is associated with radiographic structural progression [8], discriminates people with knee OA from those without knee OA [9], and is more responsive to change over time than is radiographic assessment [10]. In each of these studies, the femur (defined as the whole of the lateral and medial femoral condyles) had greater discrimination and responsiveness to change than did the tibia or patella. The femoral bone shape (“*B*-score”) metric is a form of statistical *z*-score that represents the position of a femoral bone shape along a shape vector from a non-OA knee shape (origin) toward an OA knee shape (positive direction). Non-OA and OA knees used to define this 3-dimensional (3D) shape vector were categorized using centrally read and adjudicated Kellgren-Lawrence grading [11].

Bowes et al. demonstrated in a large observational cohort of over 4500 subjects’ knees from the Osteoarthritis Initiative (OAI) that MRI-measured *B*-score produced logistic regression models for clinically important outcomes that were very similar in terms of predictive validity to those using categorical Kellgren-Lawrence grading (KLG), the conventional radiographic standard for OA diagnosis. These data provide construct validity for this new, continuous scalar measurement. In addition, Bowes et al. showed that bone shape is directly associated with the risk of clinical outcome measures such as knee pain, functional deficit, and joint failure, as indicated by total joint replacement, with only small effect sizes from adjusting models for potential covariates such as age, sex, ethnicity, body mass index (BMI), alignment, previous knee surgery, non-steroidal anti-inflammatory drug (NSAID) use, and smoking status [11]. These findings support femoral *B*-score as a structural endpoint in clinical trials of disease-modifying osteoarthritis drugs (DMOADs) and prompted the present analysis.

## Methods

Subjects between the ages of 25 and 75 years with bilateral patellofemoral (PF) knee OA were enrolled at 15 sites across the United States. Written informed consent was obtained prior to study enrollment. All subjects were screened by MRI, centrally read, to confirm structural inclusion criteria: bilateral PF cartilage defects (ICRS grade 2–3) with intact menisci. One knee was randomly assigned to receive 4 weekly injections of TPX-100, while the contralateral knee received placebo (saline) injections that were identical in appearance and viscosity. Investigators, subjects, sites, and sponsor were blinded to treatment assignment.

MRI images were acquired at baseline, 6 and 12 months with 1.5-T clinical MRI scanners using a sagittal T1-weighted 3D SPGR, FLASH, or FFE sequence with fat saturation or water excitation. An identical scanner and knee coil were used for baseline and follow-up measurements of each participant. Acquisition parameters were as follows: contiguous slice thickness 1.5 mm, in-plane resolution of 0.31 mm, repetition time 17 ms, echo time 7 ms, and flip angle 15°. Identical acquisition parameters were used at baseline and follow-up.

In the present study TPX-100-5, MRIs from all subjects (*n* = 93) who received, as randomly assigned, 4 weekly injections of TPX-100 (200 mg/injection) in one knee (index) and placebo in the contralateral knee (control) with at least one follow-up MRI were eligible for inclusion. Image quality was assessed centrally and blind to clinical data and treatment assignment. Quality was sufficient to include bilateral knee images from 78 of the 93 subjects (84%) analyzed in study TPX-100-1 (see Fig. 1; CONSORT diagram).

Cartilage measurement was performed centrally, blind to treatment assignment and clinical data, at a single center (Chondrometrics GmbH, Freilassing, Germany). The subchondral bone and cartilage surface areas of the medial and lateral tibia and medial and lateral weight-bearing central femoral condyles were traced manually, excluding cartilage cover of osteophytes [12]. Quantitative measures of cartilage, including mean cartilage thickness averaged over the total area of subchondral bone, were computed using Chondrometrics software [13]. Mean cartilage thickness over the total area of subchondral bone for the medial tibiofemoral compartment was computed by summing the values of the medial tibia and those of the weight-bearing medial femoral condyle, and for the lateral tibiofemoral compartment by summing the values of the lateral tibia and those of the lateral weight-bearing femoral condyle. Definitions of the tibiofemoral subregions as well as measurement reliability (test-retest reproducibility with repositioning of the joint between acquisitions) have been published previously [13].

**Fig. 2:**
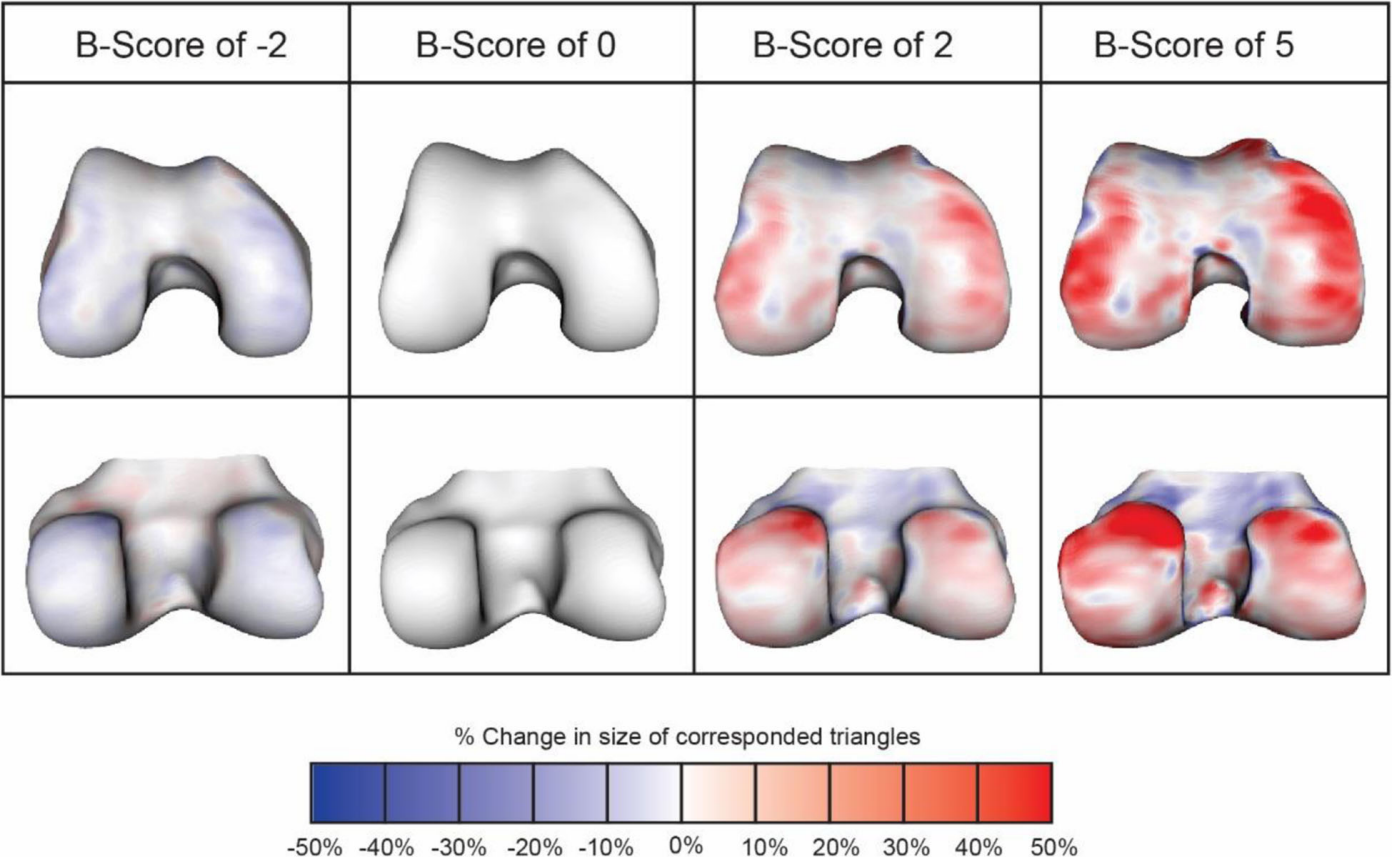
Change in shape for the anterior femur (top row) and posterior femur (bottom row), for various *B*-scores. Red indicates where there is an increase in size (locally calculated, based on anatomically corresponded triangles from the shape model), blue indicates decrease in size (locally); scale shows percentage in area size change of each triangle. Change tends to be greatest around the edge of the cartilage plate (osteophyte region), but it also occurs in central subchondral regions where the bone flattens out. Adapted from Bowes et al. [11]

Femur bone surfaces were automatically segmented using active appearance models (AAMs) at a single center (Imorphics Ltd, Manchester, UK). An AAM is a type of statistical shape model trained using machine learning to search images. AAMs have proven to be a successful supervised machine learning method that can produce a segmented knee bone surface with sub-millimeter accuracy [9, 14]. AAMs were constructed using a training set of 96 knee DESS sequence MRIs that were selected to provide examples of OA across KLG grades from subjects enrolled in the OAI [15]. The performance of the AAM has been tested with various other MRI sequences and slice thicknesses. Mean automated segmentation accuracy using MRI FLASH sequences is − 0.0009 mm, with ± 95th percentiles of error of + 0.34/− 0.43 mm, where a positive error represents the automated surface being outside the reference surface [16]. The construction of an AAM produces a “shape space,” spanned by the set of principal components used to describe the training set of examples. Within this shape space, an “OA vector” is defined as the line that passes through the mean shape of two populations: a population with OA (OA group, defined as all knees with KLG ≥ 2 over 4 years of follow-up) and a population without OA (non-OA group, defined as knees with KLG of 0 in the same period). Distances along the femur OA vector are termed “*B*-score”, with the origin (*B*-score = 0) defined as the mean shape of the non-OA group for each sex, and 1 unit defined as 1 standard deviation of the non-OA group along the OA vector (positive values toward the OA Group). Each parameterized femur bone shape was projected orthogonally onto the OA vector to specify the corresponding *B*-score value [11]. Representative examples of differences in femur bone shape at various *B*-scores and a heat map of the areas that change most with increasing *B*-score are shown in Fig. 2.

*B*-score changes over 6 and 12 months were graphically compared with the 12-month trajectories of “non-progressor” and “progressor” *B*-score groups from OAI data. The non-progressor group was defined as all of those who did not change by more than 95% of the smallest detectable difference (SDD) using the slope of change measured over 4 years. The average slope over this 4-year period was about 0.04 *B*-score units per annum. The progressor group was defined as all of those who *did* change by more than 95% of the SDD using the slope of change measured over slope over 4 years. The average slope over this 4-year period was about 0.24 *B*-score units per annum. SDD was calculated as the 95% limit of agreement between the first and second image measurements from test-retest data, using the Bland-Altman method [11].

In study TPX-100-5, paired Student’s *t*-tests were used to compare *B*-score change from baseline between the knee receiving IA placebo (control) or TPX-100 (index) at 6 months and 12 months. For each knee (index, control) at 6 months and 12 months, the Pearson coefficients of the changes from baseline between the femur *B*-score and cartilage thickness of tibiofemoral variables were estimated. The two-sided *p*-values and 95% confidence intervals from the test of the null hypothesis (that the true correlation coefficient is equal to zero) were also computed. Statistical significance was set at *P* < 0.05. The data analysis for this paper was generated using SAS software version 9.

## Results

Characteristics of the cohorts from Study TPX-100-1 and the present Study TPX-100-5 were as follows:

- All per protocol (PPT) subjects: TPX-100-1, *N* = 93
  - Mean age, 58.4 (95% C.I. 56.4, 60.4)
  - Sex, 38 males, 55 females (59.1% females)
  - Mean BMI, 30.4 (95% C.I. 29.1, 31.7)
- Evaluable *B*-score analyzed cohort: TPX-100-5, *N* = 78
  - Mean age, 58.4 (95% C.I. 56.2, 60.6)
  - Sex, 30 males, 48 females (61.5% Females)
  - Mean BMI, 30.9 (95% C.I. 29.5, 32.3)

Relative proportions of tibiofemoral ICRS grades of index and control knees of the subjects, determined as the maximum degree of cartilage defect observed in the tibiofemoral knee compartment in study TPX-100-1 and the current study, TPX-100-5, are provided in Table 1.

**Table 1:** ICRS grades of index and control knees of the subjects analyzed in TPX-100-1 and TPX-100-5 studies, respectively at baseline. ICRS grade in each knee was determined as the maximum degree of cartilage defect observed in tibiofemoral knee compartment. Subjects were screened clinically and with MRI, with inclusion criteria applied for patellofemoral OA severity (grades 2–3) only, and agnostic for OA severity in other joint compartments. One control knee failed ICRS grade measurement

ICRS grade	TPX-100-1 (***N*** = 93)		TPX-100-5 (***N*** = 78)	
	Index	Control	Index	Control
4	37% (34)	37% (34)	35% (27)	34% (26)
3	25% (23)	21% (19)	27% (21)	19% (15)
2	19% (18)	21% (19)	18% (14)	22% (17)
1	0% (0)	0% (0)	0% (0)	0% (0)
0	19% (18)	21% (20)	20% (16)	25% (19)
Total	100% (93)	100% (92)	100% (78)	100% (77)

Proportions of *B*-scores of index and control knees of the subjects analyzed in TPX-100-5 study are provided in Table 2.

**Table 2:** Femoral *B*-scores of index and control knees of the subjects analyzed in TPX-100-5 study at baseline. Mean *B*-scores of index and control knees at baseline were the same. A *B*-score of 1.48 corresponds to the mean of the range for a KLG of 2 [11]

Baseline	TPX-100-5 (***N*** = 78)	
*B*-score	Index	Control
< − 2 to − 1	4% (3)	1% (1)
< − 1 to 0	15% (12)	19% (15)
> 0 to 1	24% (19)	24% (19)
> 1 to 2	23% (18)	23% (18)
> 2 to 3	12% (9)	10% (8)
> 3 to 4	15% (12)	10% (8)
> 4 to 5	0% (0)	9% (7)
> 5 to 6	3% (2)	1% (1)
> 6 to 7	1% (1)	1% (1)
> 7 to 8	3% (2)	0% (0)
Total	100% (78)	100% (78)
Mean	1.48	1.48

**Fig. 3:**
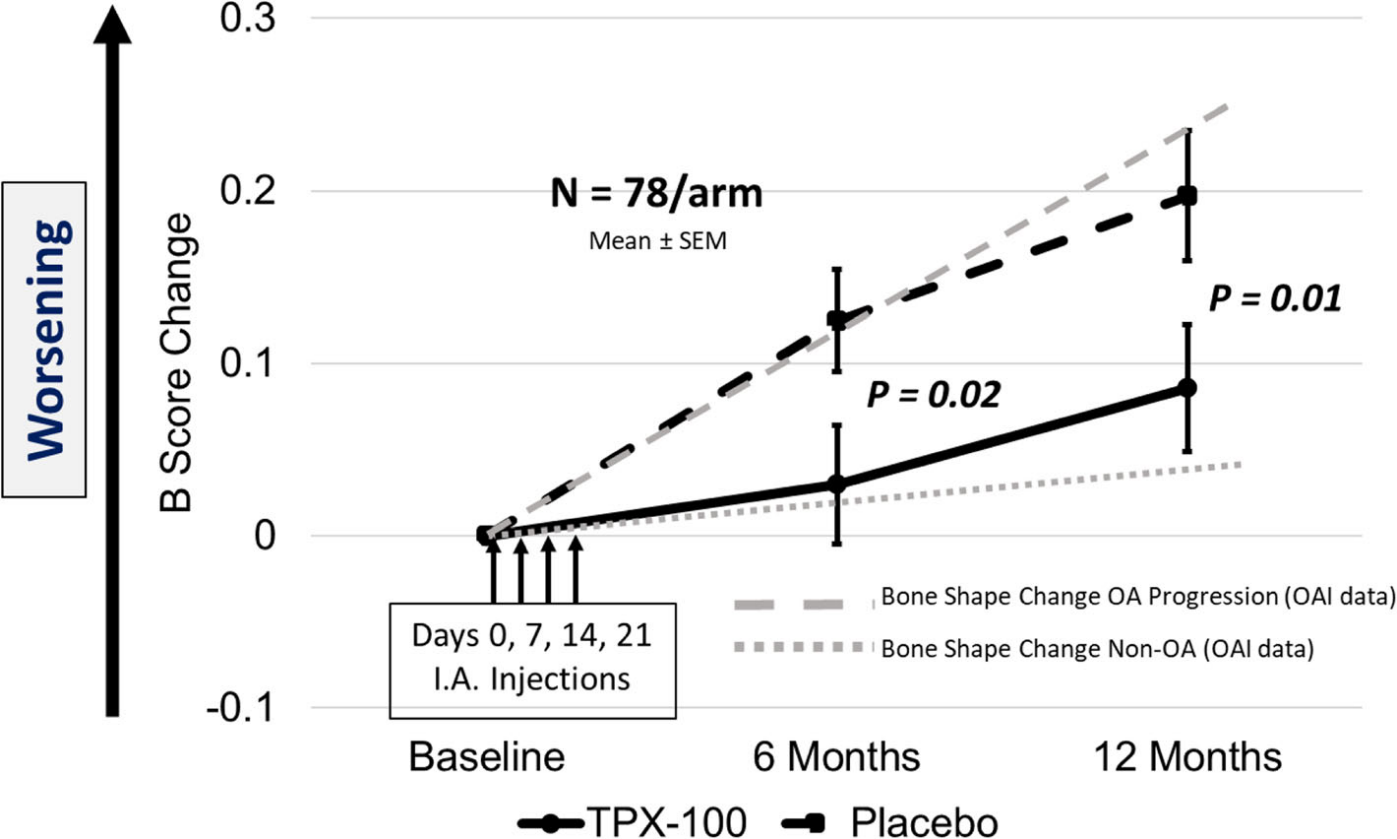
Femoral bone shape changes in TPX-100-treated knees and placebo-exposed knees in bilateral OA subjects and their comparison with historical bone shape change from the Osteoarthritis Initiative (OAI). All error bars represent standard errors of the mean

Comparison of the 78 evaluable TPX-100-treated knees demonstrated a statistically significant decrease in *B*-score change in the femur compared with placebo-exposed knees at 6 months, 0.0298 (95% C.I. − 0.037, 0.097) vs 0.1246 (95% C.I. 0.067, 0.182) (*P* = 0.02), and at 12 months, 0.0856 (95% C.I. 0.013, 0.158) vs 0.1969 (95% C.I. 0.123, 0.271) (*P* = 0.01). Graphical comparisons of *B*-score change at 6 months with progressor and non-progressor groups from OAI data showed *B*-score trajectories in TPX-100-treated knees that were strikingly similar to those of OA non-progressors, and, among placebo-exposed knees, *B*-score trajectories that were similar to OA progressors (Fig. 3). The cohort including all knees, regardless of baseline ICRS grade, demonstrated *B*-score changes significantly different in favor of TPX-100 treatment compared with controls at both 6 (*p* = 0.02) and 12 months (*p* = 0.01). Knees more severely affected (ICRS grades 3–4) were significantly different in favor of TPX-100 at 6 months (*p* = 0.01), with a non-significant trend (= 0.09) at 12 months (Table 3).

**Table 3:** Reduction of pathological bone shape change (*B*-score) by TPX-100 treatment tends to improve in the knees with higher ICRS grades at baseline. 6 and 12-month *B*-score changes of index and control knees were compared among the baseline tibiofemoral ICRS grade cohorts. Data of baseline ICRS grade ≥ 0 (i.e., all knees) are the same as those shown in Fig. 3 and comparison between index and control knees was by paired *t*-test. Because index and control knees in the higher baseline ICRS grade cohorts did not necessarily belong to the same subjects, the comparisons in these three cohorts are by unpaired *t*-tests

Baseline tibiofemoral ICRS grade	Mean baseline *B*-score (number of knees)		*B*-score difference between index and control knees			
			6 months		12 months	
	Index	Control	Difference	*p*	Difference	*p*
≥ 0 (All)	1.48 (78)	1.48 (78)	0.09	***0.02***	0.11	***0.01***
≥ 2	1.76 (62)	1.78 (57)	0.08	*0.09*	0.11	*0.07*
≥ 3	2.15 (48)	2.39 (41)	0.16	***0.01***	0.13	*0.09*
4	2.84 (27)	3.01 (26)	0.23	***0.01***	0.16	0.14

**Fig. 4:**
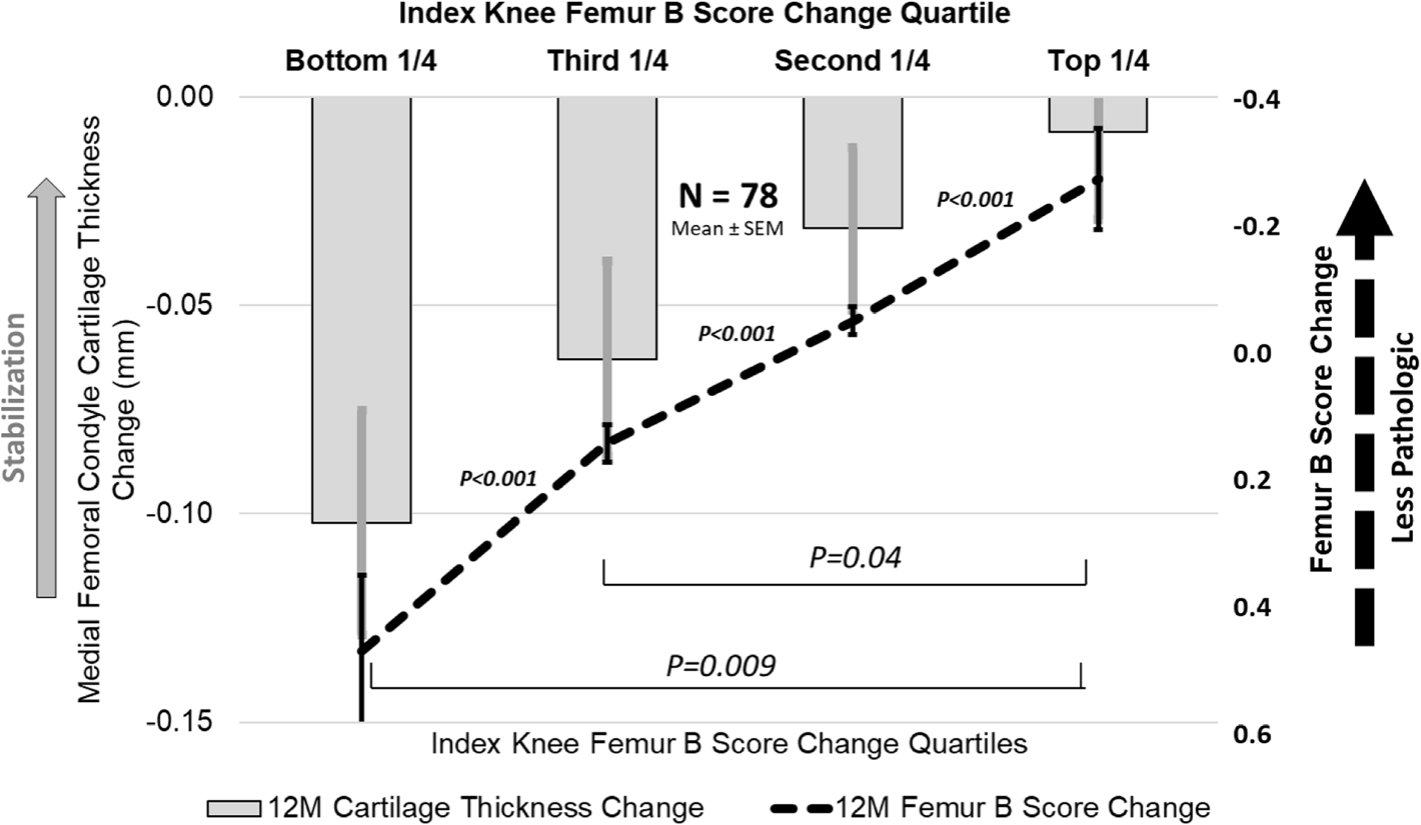
12-month femoral bone shape stabilization is associated with medial femoral condyle cartilage thickening/stabilization in the index knee. Subjects are stratified by *B*-score change quartiles. All error bars are standard errors of the mean

Analysis of quartiles of index knee *B*-score change at 12 months showed a statistically significant association between less cartilage thickness loss, particularly in the medial femoral condyle, and lower positive (worsening) *B*-score change (Fig. 4).

In TPX-100-treated knees, correlation analyses revealed a statistically significant association between *B*-score change and medial femoral condyle cartilage thickness change at 6 months and a statistically significant association between *B*-score change and cartilage thickness change in the entire femoral condyle and entire tibiofemoral (TF) compartments at 12 months (*P* < 0.05; Table 4). There was also a significant association between *B*-score change and cartilage thickness change in the medial femoral condyle and medial TF compartments at 12 months (*P* < 0.005; Table 4).

**Table 4:** Pearson correlations between femur bone shape stabilization and tibiofemoral cartilage thickening/stabilization in TPX-100-treated knee

Change period	Knee compatment	*n*	Correlation coefficient	*p-*value
Baseline to 6 months	Entire femoral condyle	78	− 0.206	0.07
	Medial femoral condyle	78	− 0.265	***0.02***
	Entire TF	78	− 0.193	0.09
	Medial TF	78	− 0.212	0.06
Baseline to 12 months	Entire femoral condyle	78	− 0.303	***0.007***
	Medial femoral condyle	78	− 0.329	***0.003***
	Entire TF	78	− 0.296	***0.009***
	Medial TF	78	– 0.320	***0.004***

Cartilage thickness change in the lateral femoral condyle and lateral TF compartments did not show an association with *B*-score changes at either 6 or 12 months. There was little or no association between *B*-score change and cartilage thickness change in control knees, with the exception of the medial femoral condyle at 12 months (*p* = 0.03) (Table 5).

**Table 5:** Pearson correlations between femur bone shape stabilization and tibiofemoral cartilage thickening/stabilization in control knee

Change period	Knee compartment	*n*	Correlation coefficient	*p*-value
Baseline to 6 months	Entire femoral condyle	78	− 0.068	0.55
	Medial femoral condyle	78	− 0.053	0.65
	Entire TF	78	0.043	0.71
	Medial TF	78	0.011	0.93
Baseline to 12 months	Entire femoral condyle	78	− 0.174	0.13
	Medial femoral condyle	78	− 0.246	***0.03***
	Entire TF	76	0.011	0.92
	Medial TF	77	− 0.085	0.46

## Discussion

In this study, we measured femoral bone shape using the *B*-score metric that describes changes along a vector tracing a linear path from a normal, non-osteoarthritic state to the pathological shape of the osteoarthritic knee. Pathological bone shape change is characterized by the broadening and flattening of the femoral condyles and tibial plateaus and the growth of osteophytes. The vector is defined by KLG = 0 at its origin and KLG ≥ 2, where a higher *B*-score is more radiographically pathologic, and, as demonstrated in previous work, is associated with progressive knee OA. Knees treated with IA TPX-100 had a bone shape change at 6 and 12 months similar to that of knees classified as KLG = 0. This was substantially different from the trajectory of control knees, which was similar to that of knees classified as KLG ≥ 2. There were no significant baseline group differences in any demographic, clinical, or MRI measures. The change in slope among treated knees compared with control knees from baseline to 6 months was more striking than that from six months to the end of the study, suggesting that a second series of injections would be a reasonable consideration in planning the next clinical trial.

These data represent, to our knowledge, the first report of an investigational agent demonstrating a significant treatment difference compared with placebo on pathologic bone shape (*B*-score) change in the knee. A previous study of the novel Cathepsin K Inhibitor MIV-711 demonstrated a reduction of pathological bone *surface area* change in subjects with knee OA at 6 months, with no treatment differences in clinical outcomes [17]. While clinically meaningful, treatment-related differences in *B*-score changes have not been defined, the difference in the rate of *B*-score change between TPX-100-treated knees and placebo-exposed knees was notable, particularly in the most severely osteoarthritic knees, suggesting a slowing of the OA-related bone pathology associated with OA progression and predictive of joint failure. In addition, treatment-associated reductions in *B*-score correlated significantly with reduced cartilage thickness loss in medial and total tibiofemoral cartilage in TPX-100-treated knees at 12 months.

TPX-100 acts on cells committed to hard tissue lineage, viz. bone, cartilage, or dentin [2–4, 18]. Since TPX-100 has been demonstrated to have positive effects on both cartilage and bone formation, activities observed in this clinical trial may be due to both activities. Based on preliminary in vitro data, the putative mechanism of action of TPX-100 is via integrin binding via the RGD sequence found in TPX-100 (unpublished data).

There were limitations in this study. TPX-100-5 was a retrospective analysis of the subset of Study TPX-100-1 subjects including those with tibiofemoral OA, mild to severe, in addition to PFOA, whose MRI images were qualified for bone shape analyses. However, the statistical analysis plan for the study was finalized prior to image qualification and analysis, and all analyses were performed blind to time point, treatment assignment, and all clinical information. As bone-shape analysis was not part of the original (TPX-100-1) study plan, the MR image sequence used was designed for manual cartilage segmentation and therefore was not optimal for automated bone segmentation and shape analysis, for which thinner, 0.75-mm slices would have been preferred. Nevertheless, all the image data that could be utilized were included in the analysis without any specific subject selection. The severity of OA in the subject population was defined by using MRI-based cartilage defect (ICRS) scoring after clinical inclusion/exclusion criteria were met. ICRS grading was performed by central readers blind to clinical information in order to establish a baseline of structural severity and to exclude significant meniscal pathology and synovitis, per study exclusion criteria.

## Conclusions

These results of structural imaging in TPX-100-treated knees compared with placebo-exposed knees, in combination with previously reported robust improvements in established clinical outcomes (as measured by WOMAC and KOOS scores), support the potential of TPX-100 as a candidate disease-modifying drug in knee OA.

## Supplementary Information

**Additional file 1.** AC-100 Promotes Cartilage Defect Repair In Vivo and Chondrocyte Differentiation and Function In Vitro

## Data Availability

Osteoarthritis Initiative (OAI) source data are available from https://data-archive.nimh.nih.gov/oai/. The datasets generated and/or analyzed within this publication are available from the corresponding author on reasonable request.

## Abbreviations

AAM: Active appearance models
BMI: Body mass index
CONSORT: *Consolidated standards of reporting trials*
DESS: Double echo steady state
DMOAD: Disease-modifying osteoarthritis drug
FFE: Fast field echo (Philips)
FLASH: Fast low-angle shot (Siemens)
IA: Intra-articular
ICRS: International cartilage repair society
KLG: Kellgren-Lawrence grading
*KOOS*: Knee injury and osteoarthritis outcome score
MEPE: Matrix extracellular phosphoglycoprotein
MOAKS: MRI osteoarthritis knee score
MRI: Magnetic resonance imaging
NRS: Numerical rating scale
OA: Osteoarthritis
OAI: Osteoarthritis Initiative
PF: Patellofemoral
PFOA: Patellofemoral osteoarthritis
PPT: Per protocol
SAS: Statistical analysis software
SDD: Smallest detectable difference
SIBLING: Small integrin-binding ligand N-linked glycoprotein
SPGR: Spoiled gradient-recalled (GE)
TF: Tibiofemoral
3D: Three dimensional
WOMAC: Western Ontario and McMaster universities osteoarthritis index

## References

[1] Hopwood B, Tsykin A, Findlay DM, Fazzalari NL (2007). Microarray gene expression profiling of osteoarthritic bone suggests altered bone remodelling, WNT and transforming growth factor-β/bone morphogenic protein signalling. Arthritis Res Ther.

[2] Hayashibara T, Hiraga T, Yi B, Motoyoshi N, Kumagai Y, Nishimura R (2004). A synthetic peptide fragment of human MEPE stimulates new bone formation in vitro and in vivo. J Bone Miner Res.

[3] Nagel DE, Khosla S, Sanyal A, Rosen DM, Kumagai Y, Riggs L (2004). A fragment of hypophospatemic factor, MEPE, requires inducible cyclooxygenase-2 to exert potent anabolic effects on normal human marrow osteoblast precursors. J Cell Biochem.

[4] Middleton-Hardie CA, Aberman H, Simon T, Alliston T, Mortazavi A, Rosen DM. AC-100 Promotes Cartilage Defect Repair In Vivo and Chondrocyte Differentiation and Function In Vitro [abstract]. Proceedings of the 56th Annual Meeting of the Orthopedic Research Society, poster # 0974. Presented March 6, 2010. New Orleans, LA

[5] McGuire D, Lane N, Segal N, Metyas S, Barthel H, Miller M (2018). TPX-100 leads to marked, sustained improvements in subjects with knee osteoarthritis: pre-clinical rationale and results of a controlled clinical trial [abstract]. Osteoarthr Cartil.

[6] McGuire D, Segal N, Metyas S, Barthel H, Miller M, Rosen D, et al. Intra-articular TPX-100 in knee osteoarthritis: robust functional response at 6 and 12 months is associated with increased tibiofemoral cartilage thickness [abstract L16]. Arthritis Rheumatol. 2018;70(suppl 10):3405-3406.

[7] Neogi T, Bowes MA, Niu J, De Souza KM, Vincent GR, Goggins J, et. al. Magnetic Resonance Imaging-Based Three-Dimensional Bone Shape of the Knee Predicts Onset of Knee Osteoarthritis: Data From the Osteoarthritis Initiative*.* Arthritis Rheum 2013;65(8):2048-2058. doi: 10.1002/art.37987. 10.1002/art.37987 PMC3729737 23650083

[8] Barr AJ, Dube B, Hensor E, Kingsbury S, Peat G, Bowes M, et. al. The relationship between three-dimensional knee MRI bone shape and total knee replacement—a case control study: data from the Osteoarthritis Initiative. Rheumatology 2016; 55:1585–1593. doi: 10.1093/rheumatology/kew191. 10.1093/rheumatology/kew191 PMC4993955 27185958

[9] Bowes MA, Vincent GR, Wolstenholme CB, Conaghan PG (2013). A novel method for bone area measurement provides new insights into osteoarthritis and its progression. Ann Rheum Dis.

[10] Hunter D, Nevitt M, Lynch J, Kraus VB, Katz JN, Collins JE, et. al*.* Longitudinal validation of periarticular bone area and 3D shape as biomarkers for knee OA progression? Data from the FNIH OA Biomarkers Consortium. Ann Rheum Dis 2016; 75:1607–1614. doi: 10.1136/annrheumdis-2015-207602. 10.1136/annrheumdis-2015-207602 26483253

[11] Bowes MA, Kacena K, Alabas OA, Brett A, Dube B, Bodick N, et.al. Machine-learning, MRI bone shape and important clinical outcomes in osteoarthritis: data from the Osteoarthritis Initiative. Ann Rheum Dis. 2020;80(4):502-508. doi: 10.1136/annrheumdis-2020-217160. 10.1136/annrheumdis-2020-217160 PMC7958089 33188042

[12] Eckstein F, Ateshian G, Burgkart R, Burnstein D, Cicuttini F, Dardzinski B (2006). Proposal for a nomenclature for Magnetic Resonance Imaging based measures of articular cartilage in osteoarthritis. Osteoarthr Cartil.

[13] Wirth W, Eckstein F (2008). A technique for regional analysis of femorotibial cartilage thickness based on quantitative magnetic resonance imaging. IEEE Trans Med Imaging.

[14] Williams TG, Holmes A, Waterton J, Maciewicz R, Hutchinson CE, Moots R (2010). Anatomically corresponded regional analysis of cartilage in asymptomatic and osteoarthritic knees by statistical shape modelling of the bone. IEEE Trans Med Imaging.

[15] Hunter DJ, Nevitt M, Lynch J, Kraus V, Katz JN, Collins J, et. al*.* Preliminary assessment of predictive validity of periarticular bone area and shape markers in knee OA. Osteoarthr Cartil 2013; 21: S175. doi: 10.1016/j.joca.2013.02.376.

[16] Bowes MA, Lohmander LS, Wolstenholme CBH, Vincent GR, Conaghan PG, Frobell RB (2019). Marked and rapid change of bone shape in acutely ACL injured knees – an exploratory analysis of the Kanon trial. Osteoarthr Cartil.

[17] Conaghan PG, Bowes MA, Kingsbury S, Brett A, Guillard G, Rizoska B, et. al*.* Disease-Modifying Effects of a Novel Cathepsin K Inhibitor in Osteoarthritis. Ann Intern Med 2020;172(2):86-95. doi: 10.7326/M19-0675. 10.7326/M19-0675 31887743

[18] Liu H, Li W, Gao C, Kumagai Y, Blacher RW, DenBesten PK (2004). Dentonin, a Fragment of MEPE, Enhanced Dental Pulp Stem Cell Proliferation. J Dent Res.

