# Supplementary material for: Study TPX-100-5: intra-articular TPX-100 significantly delays pathological bone shape change and stabilizes cartilage in moderate to severe bilateral knee OA
Source: Arthritis Res Ther. 2021 Sep 17;23:242. doi: 10.1186/s13075-021-02622-8 (PMC8447757; doi:10.1186/s13075-021-02622-8)
Supplement: Supplementary file 1 — Additional file 1. AC-100 Promotes Cartilage Defect Repair In Vivo and Chondrocyte Differentiation and Function In Vitro [file 13075_2021_2622_MOESM1_ESM.pdf]

●AC-100 Promotes Cartilage Defect Repair In Vivo and Chondrocyte Differentiation and Function In Vitro

+[1]Middleton-Hardie, C A; [2]Aberman, H; [2]Simon, T, [3]Alliston, T, [3]Mortazavi, A; [1]Rosen, D M
+[1]Acologix Inc., Hayward, CA, [2]Applied Biological Concepts, Los Alamitos, CA,
[3]Department of Orthopaedic Surgery, University of California San Francisco, CA
david.rosen@acologix.com

ABSTRACT INTRODUCTION:

Knee cartilage damage is common in the general population. This damage can be due to the degenerative processes of osteoarthritis, RA or from physical injury. Cartilage damage from physical injury is also correlated with accelerated development of OA features such as joint space narrowing, knee pain and collagen II breakdown. Unlike bone, cartilage is not able to effectively repair itself. Once degenerative processes have begun there is little that can be done to reverse it.

AC-100 is a synthetic peptide the sequence of which is derived from the central 23aa region of human extracellular matrix phosphoglycoprotein (MEPE). The sequence is highly conserved among species and contains an RGD integrin-binding motif and an SGDG glycosaminoglycan-attachment motif. AC-100 has potent anabolic activity on osteoblast and odontoblast precursor cells in vitro and has shown tissue-specific activity on bone and dental defects to promote healing in vivo.

Chondrocytes, like osteoblasts and odontoblasts derive from mesenchymal progenitor cells. Many growth factors that regulate differentiation of one mesenchymally-derived cell lineage also regulate others. This report describes the investigation of AC-100 activity to promote differentiation or function of cells in the chondrocyte lineage as well as activity in vivo in a cartilage defect model

METHODS:

Human mesenchymal stem cells (hMSCs, Lonza Inc) were expanded in monolayer culture. Pellet cultures (approximately 2.5×10^5 cells/pellet) were created by centrifugation at 500g for 5 minutes. All pellets were grown for 21 days in chondrogenic media containing insulin, transferrin, sodium selenite and 4μM TGFβ-3 in the absence or presence of AC-100 (0.4, 1 or 4μM). New media and drug were added every other day. RNA was harvested from cultured pellets, after which cDNA was generated by reverse transcription. In each condition the expression of chondrocyte marker genes including aggrecan, collagen II and collagen X was evaluated using established primer sets by quantitative PCR. Gene expression was normalized to expression of ribosomal protein L19 and is expressed as fold change relative to pellets grown in the absence of AC-100. Pellets harvested for histological analysis were fixed in formalin and processed for paraffin embedding. Six micron sections were prepared and stained with Safranin O/Fast Green to visualise proteoglycan content and localisation.

Critical sized, full-thickness cartilage defects were created in the proximal lateral trochlear sulcus of the right femora of skeletally mature female Spanish goats. The test articles (saline; AC-100 25, 125 and 250mg/application) were administered by intra-articular injection (1.5mL) immediately after closing and at 1, 2 and 3 weeks post-surgery. All animals were returned to full weight-bearing activity post-surgery. The endpoint was at 6 months where the joints were harvested and the defects scored for gross observations of healing. The bones were decalcified and processed for paraffin embedding. Five micron serial sections were taken from the centre of the defect and stained with Haematoxylin and Eosin and Safranin O/Fast Green. The slides were evaluated for parameters of cartilage repair using a scoring system developed from that of Sellers et al., 1997. Additional sections were taken 1mm from the centre towards the medial side of the defects. These sections were evaluated qualitatively.

RESULTS:

After 21 days of culture in chondrogenic conditions, AC-100 significantly increased mRNA expression from genes encoding major constituents of articular cartilage matrix, collagen II and aggrecan. Relative to vehicle-treated pellets, aggrecan and collagen II expression were induced up to 3-fold by AC-100, suggesting that AC-100 can enhance chondrocyte differentiation of hMSCs. Collagen X, a marker gene of hypertrophic chondrocytes was also induced by AC-100, though the effects were less dramatic than for collagen II or aggrecan. The chondrogenic effects of AC-100 were even more apparent histologically. While very little Safranin O staining was detected in the vehicle-treated pellets, pellets treated with AC-100 (1 and 4μM) showed significant staining concentrated in the centre of the pellet. This suggests AC-100 promotes proteoglycan production, a feature of mature cartilage formation.

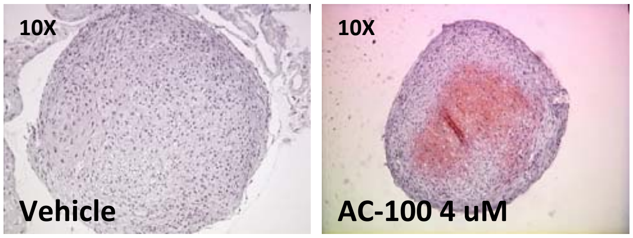

To determine if AC-100 chondrogenic activity was observed in vivo, AC-100 was administered to goat articular cartilage defects. AC-100 treated cartilage defects had better healing scores as assessed both by gross observations and histology. For the two higher doses of AC-100 there were significant improvements ($p<0.07$) in the gross parameters of healing which included amount of repair tissues, edge integration, smoothness of the surface and degree of filling as well as colour and opacity of the repair tissue. The extent and quality of repair tissue was also analysed by histology using a modified Sellers score. The defects treated with the two higher doses of AC-100 had significantly improved healing compared with vehicle ($p<0.05$).

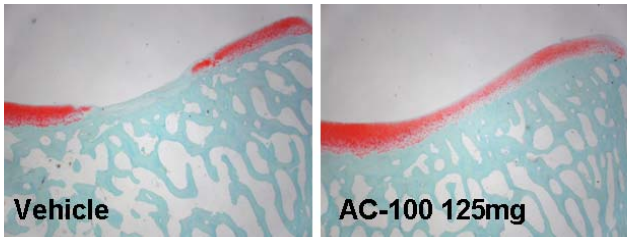

DISCUSSION:

This study investigated whether AC-100 could promote chondrocyte differentiation and function, as it does for other mesenchymally-derived cell types; osteoblasts and odontoblasts. After 21 days, AC-100 increased the expression of chondrocyte markers genes and the production of proteoglycan-rich extracellular matrix relative to vehicle-treated cells. Whether AC-100 increases the selection for the chondrogenic lineage or enhances matrix production of cells undergoing chondrogenesis remains to be determined. Dynamic studies of gene expression will elucidate the stage at which AC-100 exerts chondrogenic activity.

Importantly, the chondrogenic effects were also observed in vivo where AC-100 significantly improved healing in critical-sized defects in cartilage in a large animal model. The repair tissue formed with AC-100 treatment was mature, true cartilage with robust collagen II IHC staining and Safranin O staining indicating maturation. These results in a weight-bearing joint where the repair tissue is subjected to significant mechanical stresses are very promising.

In conclusion, these studies suggest AC-100 enhances chondrocyte differentiation and cartilage matrix production in vitro and in vivo. Therefore AC-100 represents a novel approach to repair of cartilage As AC-100 also has anabolic activity on bone, AC-100 may be an ideal agent to promote repair of cartilage as well as subchondral bone defects resulting from osteoarthritis or procedures such as microfracture or autologous chondrocyte implantation.
